# Diabetic liver injury from streptozotocin is regulated through the caspase-8 homolog cFLIP involving activation of JNK2 and intrahepatic immunocompetent cells

**DOI:** 10.1038/cddis.2013.228

**Published:** 2013-07-04

**Authors:** T Kohl, N Gehrke, A Schad, M Nagel, M A Wörns, M F Sprinzl, T Zimmermann, Y-W He, P R Galle, M Schuchmann, J M Schattenberg

**Affiliations:** 1 I. Department of Medicine, University Medical Center of the Johannes Gutenberg University, Mainz, Germany; 2 Institute of Pathology, University Medical Center of the Johannes Gutenberg University, Mainz, Germany; 3 Department of Immunology, Duke University, Durham, NC, USA

**Keywords:** apoptosis, liver injury, JNK2, hyperglycemia, cFLIP

## Abstract

The endemic occurrence of obesity and the associated risk factors that constitute the metabolic syndrome have been predicted to lead to a dramatic increase in chronic liver disease. Non-alcoholic steatohepatitis (NASH) has become the most frequent liver disease in countries with a high prevalence of obesity. In addition, hepatic steatosis and insulin resistance have been implicated in disease progression of other liver diseases, including chronic viral hepatitis and hepatocellular carcinoma. The molecular mechanisms underlying the link between insulin signaling and hepatocellular injury are only partly understood. We have explored the role of the antiapoptotic caspase-8 homolog cellular FLICE-inhibitory protein (cFLIP) on liver cell survival in a diabetic model with hypoinsulinemic diabetes in order to delineate the role of insulin signaling on hepatocellular survival. cFLIP regulates cellular injury from apoptosis signaling pathways, and loss of cFLIP was previously shown to promote injury from activated TNF and CD95/Apo-1 receptors. In mice lacking cFLIP in hepatocytes (flip^−/−^), loss of insulin following streptozotocin treatment resulted in caspase- and c-Jun N-terminal kinase (JNK)-dependent liver injury after 21 days. Substitution of insulin, inhibition of JNK using the SP600125 compound *in vivo* or genetic deletion of the mitogen-activated protein kinase (MAPK)9 (JNK2) in all tissues abolished the injurious effect. Strikingly, the difference in injury between wild-type and cFLIP-deficient mice occurred only *in vivo* and was accompanied by liver-infiltrating inflammatory cells with a trend toward increased amounts of NK1.1-positive cells and secretion of proinflammatory cytokines. Transfer of bone marrow from rag-1-deficient mice that are depleted from B and T lymphocytes prevented liver injury in flip^−/−^ mice. These findings support a direct role of insulin on cellular survival by alternating the activation of injurious MAPK, caspases and the recruitment of inflammatory cells to the liver. Thus, increasing resistance to insulin signaling pathways in hepatocytes appears to be an important factor in the initiation and progression of chronic liver disease.

An increasing prevalence of chronic liver disease has been predicted in the next decade based on the epidemic occurrence of risk factors that are associated with the metabolic syndrome, including abdominal obesity and insulin resistance. Both in adolescents and adults, non-alcoholic steatohepatitis (NASH), the predominant manifestation of the metabolic syndrome in the liver, has become the most frequent cause of liver disease.^1, 2^ The mechanisms that contribute to liver injury in the setting of hepatic steatosis and insulin resistance remain obscure. Cellular survival and hepatic tissue homeostasis involve a delicate balance between apoptosis and proliferation. At a molecular level, an equilibrium between pro- and antiapoptotic factors controls cell death and tissue turnover. Increases in antiapoptotic factors can promote the development of transformed cells and carries the risk of malignancy, whereas predominance of proapoptotic factors can augment cell loss and promote acute liver failure, as well as increased inflammation, regeneration and malignancy.^3, 4^

The cellular FLICE-inhibitory protein (cFLIP) is a caspase-8 homolog and a critical regulator of caspase activation in hepatocytes. Hepatocyte-specific deletion of cFLIP leads to increased sensitivity from apoptosis-inducing ligands with acute liver failure.^5^ In contrast, insulin signaling controls metabolic pathways and promotes cellular proliferation and survival. Activation of insulin signaling pathways involves phosphorylation of the insulin receptor, insulin receptor substrates (IRSs) and downstream mediators that contribute to glucose homeostasis, and also regulate proliferation and cellular survival of hepatocytes.^6^ A third class of contributors that affect cellular survival of hepatocytes in the context of apoptosis signaling pathways is the family of mitogen-activated protein kinases (MAPKs). Among these, the extracellular signal-regulated kinase and the c-Jun N-terminal kinase (JNK) 1 and 2 are prominent members.^7^ In hepatocytes, prolonged phosphorylation of JNK, and especially activation of JNK2, leads to the activation of caspase-8 and the mitochondrial death pathway.^8^ Similarly, alterations in the mTOR/S6K1 signaling pathway, which is connected to insulin-dependent PI3K/Akt signaling, affect IRS and promote activation of prosurvival signaling.^9^ The overlap of cell death and metabolic and proliferative signals suggests that the underlying metabolic changes including insulin resistance and obesity influence the apoptosis sensitivity in hepatocytes.^10^

The role of immunocompetent cells in liver injury is well established, and a close link between nutrition, energy homeostasis and the immune system exists.^11, 12^ Energy-sensing pathways including the AMP-activated protein kinase are involved in the regulation of inflammatory genes and in tissue inflammation, and controls autophagy and cellular survival pathways in the liver.^13, 14, 15^ In the context of obesity, natural killer T (NKT) cells regulate the recruitment of proinflammatory cell populations and link lipid excess to inflammation.^16^ Also, inhibition of NK cells alters the recruitment of inflammatory cells to the liver and reduces liver injury from cytokines.^17^

In this study, we aimed to analyze the interactions of insulin signaling and hepatocellular cell death and survival pathways. In hepatocytes that are predisposed to cellular injury due to loss of the antiapoptotic factor cFLIP, we observed increased activation of injurious MAPK signaling in a model of hypoinsulinemic diabetes. The underlying mechanism involves activation of proinflammatory lymphocytes *in vivo*, and depletion of T and B cells prevented cellular injury. In addition, deletion of JNK2 protected the mice lacking cFLIP in hepatocytes from hypoinsulinemic liver injury. Thus, our study establishes a role for insulin (1) as a critical regulator of hepatocellular stress signaling pathways and (2) as a factor that controls hepatic inflammation, thus modulating cell death pathways in liver disease.

## Results

### The caspase-8 homolog cFLIP regulates hepatocellular apoptosis from streptozotocin-induced hyperglycemia *in vivo*

**Figure 1:**
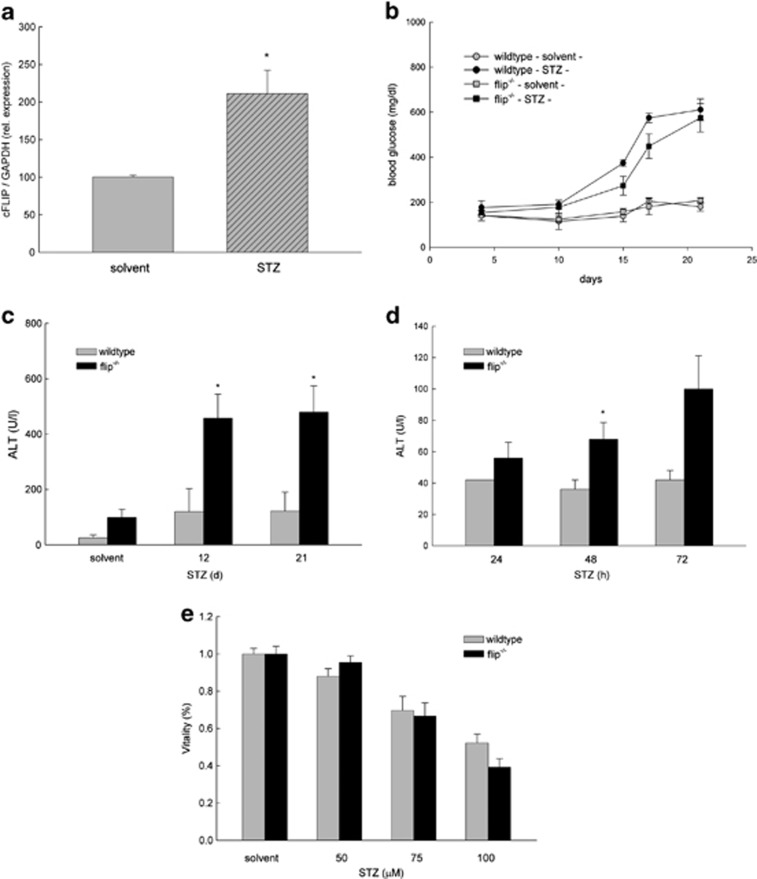
STZ-induced expression of cFLIP protects mice from liver injury. (**a**) Wt mice were treated with STZ, and the expression of cFLIP normalized to glyceraldehyde 3-phosphate dehydrogenase (GAPDH) was determined in liver tissue using real-time quantitative-polymerase chain reaction (RT-qPCR) on day 21. (**b**) Levels of serum glucose were measured at the indicated time points. (**c**–**d**) Liver injury in STZ-treated wt mice exhibiting a hepatocyte-specific deletion of cFLIP (flip^−/−^) was determined by ALT at the indicated time points. *P-value <0.05 for wt *versus* flip^−/−^. (**e**) Hepatocytes were treated *ex vivo* following collagen perfusion with increasing concentrations of STZ as indicated, and cellular viability was assessed by neutral red staining at 24 h

Streptozotocin (STZ) is a toxin that leads to inflammation and apoptosis of *β*-cells in the pancreas mimicking autoimmune diabetes. Moreover, in high concentrations, acute toxicity in extrapancreatic organs has been described.^18^ To assess the effect of hypoinsulinemia induced by repetitive low-dose STZ treatment on the expression of cFLIP, C57Bl/6 mice received 80 *μ*g/g STZ or solvent (intraperitoneally) over 5 days. We observed a 2.1-fold induction of antiapoptotic cFLIP mRNA in liver tissue on day 21 following STZ treatment (Figure 1a). To assess the role of cFLIP in hepatocytes during STZ-induced hypoinsulinemia, we used mice exhibiting a hepatocyte-specific deletion of cFLIP using the cre-loxP system under control of the albumin promoter (flip^−/−^) and compared these with cre-negative control mice (wild-type (wt)).^5^ STZ treatment resulted in hyperglycemia independent of the underlying genotype (Figure 1b). Following treatment with STZ, the levels of alanine-aminotransferase (ALT) were elevated 3.8- and 3.9-fold, respectively, in flip^−/−^ mice compared with wt on days 12 and 21 (Figure 1c). Levels of malondialdehyde (MDA), a marker of oxidative stress, doubled in response to STZ treatment; however, no significant differences between STZ-treated wt and flip^−/−^ mice were detectable (wt *versus* flip^−/−^, 3.4 *versus* 2.7 nmol/10 mg liver tissue; *P=*NS, data not shown). To differentiate STZ-related acute toxicity from hyperglycemic effects following the destruction of *β*-cells, ALT was determined following repetitive short-term treatments. Independent of hyperglycemia, flip^−/−^ mice exhibited 1.3-, 1.9- and 2.4-fold elevated levels of ALT at 24, 48 and 72 h in flip^−/−^ mice, respectively, whereas toxicity in wt mice was lower (Figure 1d). Interestingly, hepatocytes from wt and flip^−/−^ mice treated *ex vivo* following collagen perfusion exhibited comparable dose-dependent toxicity (Figure 1e, wt *versus* flip^−/−^, 52% *versus* 39% at 100 *μ*M STZ; *P=*0.06, NS).

**Figure 2:**
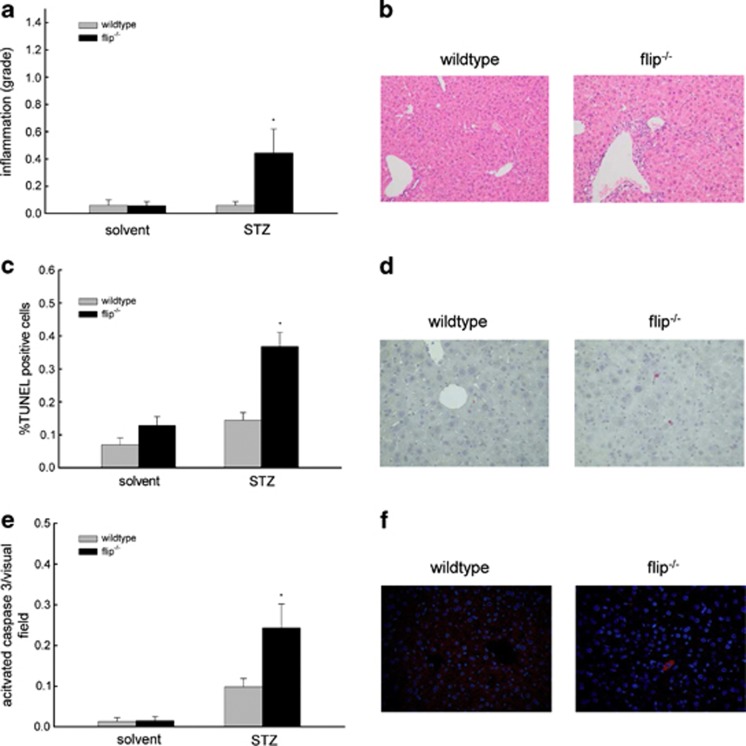
STZ treatment leads to hepatic inflammation and apoptosis in mice with cFLIP-deficient hepatocytes. (**a**) Blinded histological grading of hematoxylin and eosin (H&E)-stained liver sections using the METAVIR scoring system was performed following treatment with STZ on day 21 after treatment. (**b**) Representative histological sections are shown and depict increased lobular inflammatory cell aggregates in flip^−/−^ mice (magnification × 400). (**c**–**f**) The degree of apoptosis in the liver was assessed by quantification of TUNEL^+^ hepatocytes in percentage and immunofluorescent staining for activated caspase-3^+^ hepatocytes per visual field on day 21 following STZ treatment. (**c**) At least 10 random high-power fields (magnification × 400) were evaluated for TUNEL^+^ hepatocytes in a blinded manner. (**d**) Two representative histological sections of the STZ-treated wt and flip^−/−^ mice are shown (magnification × 400). (**e**) Activated caspase-3^+^ hepatocytes were counted in a blinded manner in at least 40 random visual fields (magnification × 200). (**f**) Two representative histological images of the STZ-treated wt and flip^−/−^ mice are shown (magnification × 200). Nucleic acid appears blue through counterstaining with 4′,6-diamidino-2-phenylindole and activated caspase-3 appears red through visualization with Alexa Fluor 555-conjugated secondary antibody

Histological analysis of liver tissue showed lobular infiltrating inflammatory cells on day 21 following STZ injections, which were significantly more pronounced in flip^−/−^ mice compared with wt mice (Figures 2a and b). Terminal dUTP nick-end labeling (TUNEL) assays demonstrated a higher degree of apoptotic hepatocytes in flip^−/−^ mice on day 21 and increased amounts of activated caspase-3 (Figures 2c–f). Levels of pro- and anti-inflammatory proteins involved in the regulation of the mitochondrial axis of the apoptosis signal, including Bid, Bcl-2 and Bcl-Xl, were not differently expressed in wt and flip^−/−^ mice and were unaffected by STZ treatment (data not shown). Thus, hepatocellular injury from STZ in mice is regulated by cFLIP and involves both acute toxicity and metabolic mechanisms that occur only *in vivo*.

### STZ-induced liver injury involves activation of the JNK pathway

**Figure 3:**
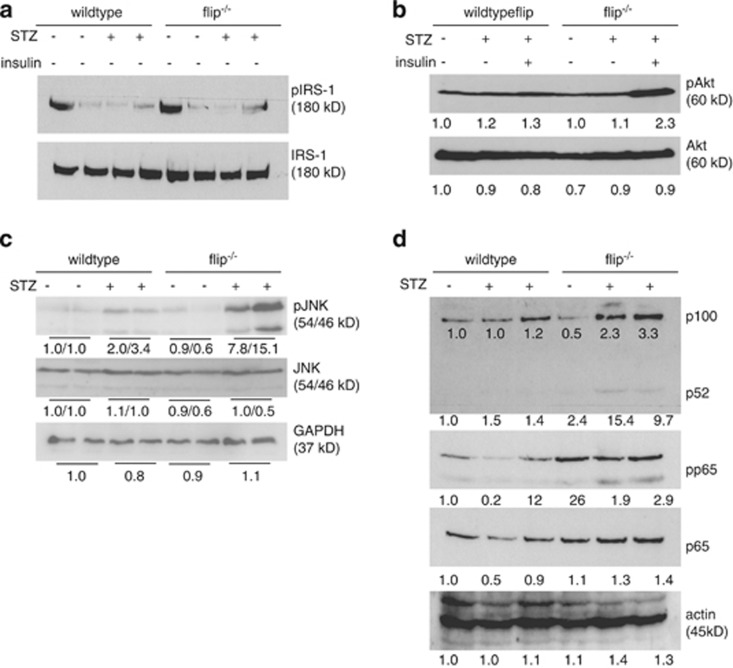
Alterations in insulin, MAPK and NF-*κ*B signaling from STZ. (**a** and **b**) Insulin signaling was assessed by immunoblotting of phospho (p)-serine and total IRS-1 and phospho- or total-Akt protein levels on day 21 following STZ treatment. Insulin was injected to correct hyperglycemia when indicated. The numbers below the panels indicate relative expression levels determined by densitometry. (**c**) Activation of JNK was assessed by immunoblotting for phosphorylated and total JNK protein. Glyceraldehyde 3-phosphate dehydrogenase (GAPDH) served as the control for equal protein loading. Densitometry values below the panels allow to calculate the ratio of phosphorylated to total JNK protein. (**d**) Expression levels of phosphorylated and total p65, p100, p52 and actin were determined by immunoblotting following treatment with STZ or solvent for 21 days as indicated. Relative expression levels are depicted as numbers below each blot

To determine the effects of hypoinsulinemia induced by STZ in hepatocytes, the insulin signaling molecules that were involved were examined. STZ treatment resulted in a significant decrease in IRS-1 serine phosphorylation in the absence of insulin irrespective of the genotype. Activation of the downstream effector Akt was not affected by STZ treatment, and injection of insulin promoted phosphorylation and activation of Akt, particularly in flip^−/−^ mice, demonstrating that insulin signaling pathways were intact in all mice (Figures 3a and b). To assess the mechanisms involved in liver injury, the levels of phosphorylated and total JNK were determined. Prolonged activation of JNK was previously shown to promote apoptotic liver injury from TNF and in a model of steatohepatitis.^8, 19^ On day 21, flip^−/−^ mice exhibited significantly increased phosphorylation and activation of the p54 and p46 JNK isoforms relative to total JNK (Figure 3c). Activation of NF-*κ*B involves alterations of RelA (p65) and NF-*κ*BB2 (p100/p52) and has been implied in inflammatory liver injury and hepatocarcinogenesis, as well as in the context of STZ-induced diabetes.^20, 21, 22^ In response to STZ treatment, the expression of p100 and p52 increased with a trend toward higher levels in flip^−/−^ mice compared with wt. Phosphorylation and expression of p65 were increased in flip^−/−^ mice compared with wt, but were unaffected by STZ treatment (Figure 3d).

### Liver injury from STZ in flip^−/−^ mice is prevented by insulin and is dependent on JNK activation

**Figure 4:**
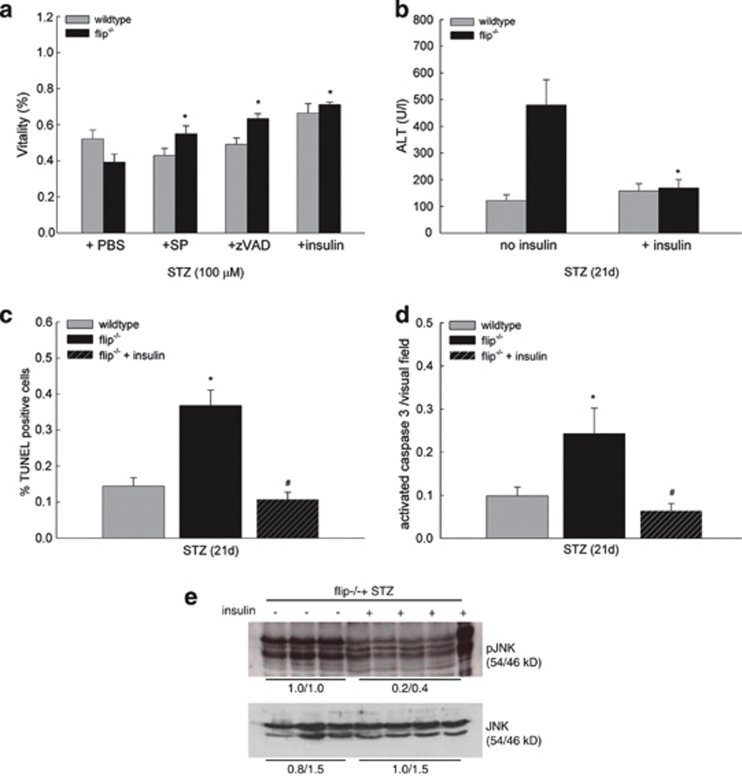
Hepatocyte cell death from STZ occurs as caspase-dependent and is ameliorated by inhibition of JNK and insulin treatment *ex vivo*. (**a**) Hepatocytes isolated by collagen perfusion of whole liver tissue of wt and flip^−/−^ mice were pretreated with PBS, SP600125, zVAD or insulin for 60 min before treatment with STZ, and cellular viability was assessed by neutral red staining at 24 h. Cellular viability is expressed relative to cells treated with phosphate-buffered saline (PBS) as negative control. *P-value<0.05 for flip^−/−^+PBS *versus* flip^−/−^+SP, zVAD or insulin. (**b**) *In vivo* liver injury was assessed in wt and flip^−/−^ mice following STZ treatment on day 21. When indicated, mice were injected with insulin to correct hyperglycemia. *P-value <0.05 for flip^−/−^±insulin. (**c** and **d**) Apoptotic cells were quantitated by (**c**) TUNEL assay and (**d**) immunofluorescence for activated caspase-3 and expressed as positive cells per visual field at × 200 magnification relative to all hepatocytes (% positive). A minimum of 10 representative visual fields were scored in a blinded manner. A *P*-value <0.05 is indicated by * for wt *versus* flip^−/−^ and by ^#^ for flip^−/−^ ±insulin. (**e**) Activation of JNK was assessed in flip^−/−^ mice following STZ treatment by immunoblotting for phosphorylated and total JNK protein on day 21. When indicated, insulin was injected to correct hyperglycemia. The numbers below the panel indicate the mean of the relative expression levels of pJNK determined by densitometry

To further substantiate the mechanisms of STZ-induced hepatocellular injury, hepatocytes from wt and flip^−/−^ mice were isolated by collagen perfusion and treated *ex vivo* as detailed above. Pretreatment with insulin significantly reduced acute cellular toxicity from STZ, and cellular viability increased 1.28-fold in wt mice and 1.81-fold in flip^−/−^ mice at 24 h (Figure 4a). Likewise, the pancaspase inhibitor Val-Ala-Asp-fluoromethylketone (zVAD) and the JNK inhibitor SP600125 decreased cellular toxcicity from STZ in flip^−/−^ hepatocytes following *ex vivo* treatment. This effect was more pronounced in flip^−/−^ hepatocytes compared with wt hepatocytes (Figure 4a). *In vivo* substitution of insulin to correct hyperglycaemia prevented the increased injury observed in flip^−/−^ mice and was accompanied by a significant reduction in the number of TUNEL-positive hepatocytes and caspase-3 activation (Figures 4b–d). In parallel, increased phosphorylation of the JNK isoforms p54 and p46 in flip^−/−^ mice following STZ treatment on day 21 was significantly ameliorated by insulin treatment (Figure 4e).

**Figure 5:**
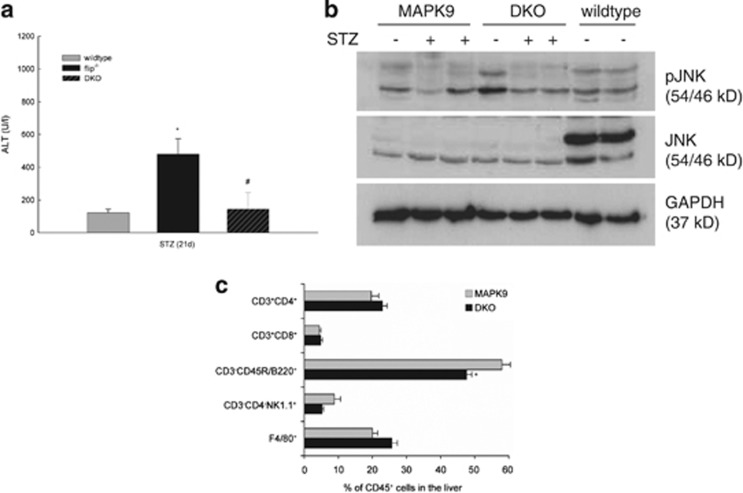
Liver injury from STZ is prevented by deletion of JNK2 in flip^−/−^ mice. (**a**) Liver injury was assessed in wt mice, flip^−/−^ mice and in mice deficient for both MAPK9 (JNK2) and cFLIP (DKO) on day 21 following STZ treatment. *P-value of <0.05 for wt *versus* flip^−/−^ and ^#^ for flip^−/−^ *versus* DKO. (**b**) Activation of JNK was assessed by immunoblotting for phosphorylated (p) and total JNK protein in JNK2-deficient (MAPK9), double-deficient JNK2 × cFLIP (DKO) and wt mice. Glyceraldehyde 3-phosphate dehydrogenase (GAPDH) served as the control for equal protein loading. (**c**) Intrahepatic immunocompetent cells derived from the liver on day 21 following treatment with STZ were compared in MAPK9 and MAPK9 × flip^−/−^ DKO animals using flow cytometry (fluorescence-activated cell sorter). Living CD45^+^ cells were gated and assigned to the following subsets: CD45^+^CD3^+^CD4^+^ and CD45^+^CD3^+^CD8^+^ for T lymphocytes, CD45^+^CD3^−^CD45/B220^+^ for B lymphocytes, CD45^+^CD3^−^CD4^−^NK1.1^+^ for NK cells and CD45^+^F4/80^+^ for macrophages. *P-value (Student's *t*-test) of <0.05 for MAPK9 *versus* DKO

To further explore the involvement of JNK in STZ-induced liver injury, we generated double-knockout (DKO) mice deficient for MAPK9 (JNK2) in all tissues and cFLIP in hepatocytes as described previously.^23^ *In vivo* liver injury from STZ was reduced by 65% in DKO mice compared with flip^−/−^ mice (Figure 5a). Phosphorylation of JNK was significantly blunted in both MAPK9 and DKO mice at baseline and following treatment (Figure 5b). Similarly, the expression of total JNK was significantly decreased and only the p46 isoform was detectable in the MAPK9 and DKO mice (Figure 5b). No difference was observed with regard to the expression or activation of the anti- or proapoptotic proteins Bcl-Xl, Bcl-2 or Bid in these mice. Similarly, levels of p100/p52, p65 and phospho-p65 were not different in MAPK9 and DKO mice (data not shown). Injury in metabolic liver disease and NASH, which is strongly correlated with impaired hepatic insulin signaling, has been attributed to the recruitment of proinflammatory, M1-polarized macrophages.^24^ To address whether STZ-induced hypoinsulinemia affects intrahepatic immune cell populations, FACS analysis of CD45^+^ liver cells was performed. On day 21 following STZ treatment, DKO mice exhibited a slight increase in the absolute number of intrahepatic CD45^+^ cells compared with MAPK9 mice (data not shown). Nevertheless, a significantly decreased amount of B lymphocytes (CD45^+^CD3^−^CD45R/B220^+^; MAPK9 *versus* DKO, 57.5% *versus* 47.2% *P*<0.05) as well as a slight reduction in the number of NK cells (CD45^+^CD3^−^CD4^−^NK1.1^+^; MAPK9 *versus* DKO, 8.7% *versus* 5.1 % *P=*NS) was observed, indicating that alterations in the intrahepatic CD45^+^ lymphocyte populations modulate hypoinsulinemic liver injury induced by STZ (Figure 5c, *n=*4).

### Loss of B and T cells in flip^−/−^ mice carrying rag-1 bone marrow reduces liver injury from STZ

**Figure 6:**
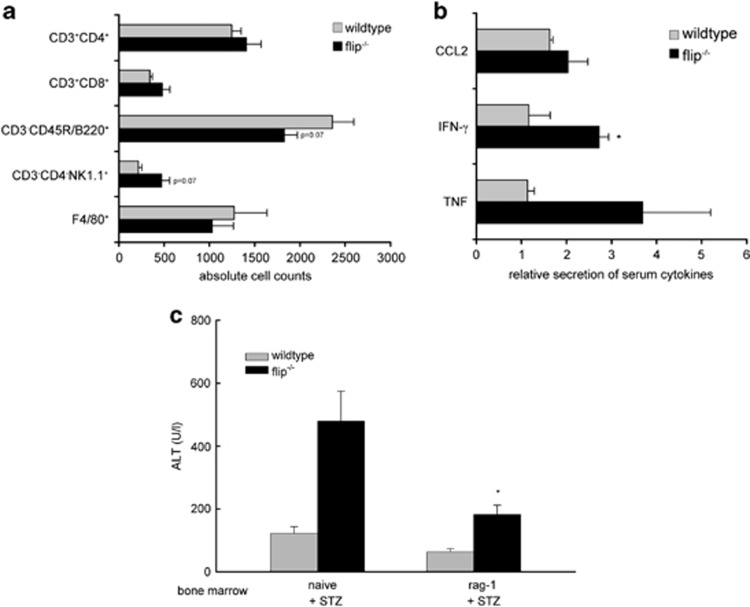
Liver injury from STZ in flip^−/−^ mice is dependent on T and B lymphocytes. (**a**) Intrahepatic immunocompetent cells derived from the liver of wt and flip^−/−^ mice were analyzed and quantified on day 21 following treatment with STZ by flow cytometry (fluorescence-activated cell sorter). Quantification of the different immune cell populations was performed by gating on living CD45^+^ cells. The cell counts of CD45^+^CD3^+^CD4^+^ and CD45^+^CD3^+^CD8^+^ for T lymphocytes, CD45^+^CD3^−^CD45R/B220^+^ for B ymphocytes, CD45^+^CD3^−^CD4^−^NK1.1^+^ for NK cells and CD45^+^F4/80^+^ for macrophages are depicted. (**b**) Levels of CCL2 (MCP-1), IFN-*γ* and TNF were measured in the serum of wt and flip^−/−^ mice on day 21 following treatment with STZ or solvent using CBA. The ratio of the cytokine amount in the serum of STZ-treated mice to control mice is shown. *P-value (Student's *t*-test) <0.05 for wt *versus* flip^−/−^ mice. (**c**) Liver injury was assessed in wt and flip^−/−^ mice on day 21 following treatment with STZ. When indicated, mice were transplanted with bone marrow derived from rag-1-deficient mice 4 weeks before treatment. *P-value (Student's *t*-test) <0.05 for flip^−/−^ mice *versus* flip^−/−^ mice transplanted with rag-1-deficient bone marrow

To assess the contribution of intrahepatic CD45^+^ lymphocyte populations, we also compared FACS analysis in wt and flip^−/−^ mice on day 21 following STZ treatment. We observed a trend toward an increased number of intrahepatic CD45^+^ cells accompanied by an accumulation of T lymphocytes (CD45^+^CD3^−^CD4^+^ and CD45^+^CD3^−^CD8^+^; wt *versus* flip^−/−^, 1589.1 *versus* 1886.2; *P=*NS) and in particular of NK cells (CD45^+^CD3^−^CD4^−^NK1.1^+^; wt *versus* flip^−/−^, 214.0 *versus* 470.5; *P=*0.07, NS) in flip^−/−^ mice compared with wt mice (Figure 6a, *n=*11). Nevertheless, as seen before in DKO mice (Figure 5c), we detected a decreased number of B lymphocytes in the liver of flip^−/−^ mice in comparison with wt mice (wt *versus* flip^−/−^, 2357.1 *versus* 1824.6; *P=*0.07, NS).

Further, we analyzed the serum of wt and flip^−/−^ mice on day 21 after treatment with STZ and appropriate controls for various cytokines and chemokines using a cytometric bead array (CBA; Figure 6b, *n=*5). Levels of CCL2 (monocyte chemotactic protein-1 (MCP-1)), IFN-*γ* and TNF were enhanced in response to STZ, but this effect was more pronounced in flip^−/−^ mice compared with wt mice, which was reflected by an increased total cytokine concentration in the serum of STZ-treated flip^−/−^ mice (wt *versus* flip^−/−^, 70.3 *versus* 104.1 pg/ml; CCL2, 1.1 *versus* 1.5 pg/ml; IFN-*γ*, 10.0 *versus* 25.4 pg/ml TNF) as well as by an enhanced ratio between the cytokine amount in the serum of STZ and solvent-treated flip^−/−^ mice. This underlines the more inflammatory phenotype of flip^−/−^ mice and the role of inflammatory cells and cytokines in STZ-induced liver injury.

To further address the potential role of lymphocytes on STZ-induced liver injury, the bone marrow of T- and B-cell-deficient rag-1 mice was transplanted in flip^−/−^ mice. Four weeks following the reconstitution of flip^−/−^ mice with rag-1-deficient bone marrow, these mice were treated with STZ, and liver injury was assessed on day 21 following STZ treatment. Interestingly, the loss of B and T lymphocytes in flip^−/−^ mice ameliorated liver injury by 47% (Figure 6c, flip^−/−^ *versus* flip^−/− rag-1 bone marrow^, 343 *versus* 182 U/l; *P*<0.05, *n=*5), whereas no effect on liver injury was observed in wt mice transplanted with bone marrow from rag-1-deficient mice (wt *versus* wt^rag-1 bone marrow^, 84 *versus* 63, *n=*7).

## Discussion

The epidemic occurrence of metabolic risk factors, including obesity and diabetes, has been predicted to lead to a dramatic increase in the prevalence of liver disease, and today NASH is considered the most frequent cause of liver injury in both adolescents and adults.^1, 2^ The pathomechanisms that promote liver cell injury in the context of these risk factors are largely unexplored and the mechanisms of disease initiation and progression are poorly defined. Hepatic tissue homeostasis involves a precisely orchestrated balance of anti- and proapoptotic factors, and mild disturbances can lead to severe organ injury.^4^ In this study, we utilize a model of low-dose toxin-induced hypoinsulinemia and hyperglycemia that is commonly used to study type I diabetes. We observed induction of the antiapoptotic protein cFLIP on day 21 in liver tissue following treatment. This implies a prolonged and lasting effect following STZ treatment in hepatocytes, and we concluded that a potential interaction of metabolic and cell death signaling pathways exists. To further investigate the role of cFLIP in this context, we used mice that exhibit a hepatocellular-specific deletion of cFLIP, which renders these mice susceptible to liver injury from apoptosis-inducing ligands.^5^ In flip^−/−^ mice liver injury, caspase activation and apoptosis were significantly enhanced compared with those in wt mice, despite similar metabolic disturbances. STZ also induced a toxic pattern of injury shortly after exposure; however, serum transaminases continued to increase even beyond the expected half-life of STZ and were significantly higher at the end of the observational period of 21 days. Interestingly, these differences in cellular injury did not occur to the same extent in *in vitro* experiments. Hepatocytes that were isolated by collagen perfusion from wt and flip^−/−^ mice and treated with STZ *ex vivo* exhibited a comparable loss of cellular viability. Thus, loss of insulin signaling and hyperglycemia rather than toxic effects contributed to persistent liver cell injury. These findings suggest a direct interaction of insulin and cell death signaling pathways in hepatocytes.

Histological examination showed increased inflammatory cells in the liver of flip^−/−^ mice on day 21. FACS analysis of the intrahepatic CD45^+^ immunocompetent cell populations also revealed a trend toward increased numbers of CD45^+^ cells, in particular NK cells. The contribution of immunocompetent cells to liver injury was validated in experiments using bone marrow-derived cells from T- and B-cell-deficient rag-1 mice.^25^ Following reconstitution of flip^−/−^ with rag-1-deficient bone marrow, liver cell injury from STZ was significantly ameloriated. Thus, hypoinsulinemia and hyperglycemia from STZ mediated changes in intrahepatic immune cell populations, which contribute significantly to liver injury. These findings are in line with previous observations on the role of immunocompetent cells promoting liver injury from apoptosis ligands and in the context of NASH through alternations in intrahepatic T-cell populations.^16, 17^ Further, we underlined the contribution of proinflammatory cytokines such as CCL2 (MCP-1), IFN-*γ* and TNF to STZ-induced liver injury.

The superfamily of MAPK constitutes a second major class of proteins that regulate liver cell survival.^7, 26^ Among these, the Akt/PI3K signaling pathway has been implicated as a prosurvival signal, and interactions with cFLIP have been observed in lymphocytes and renal epithelial cells.^27^ Inhibitory serine phosphorylation of IRS-1 decreased in the context of hypoinsulinemia following STZ treatment. Activation of Akt induced by insulin was intact and more pronounced in flip^−/−^ mice. In contrast, a strong activation of JNK was observed in flip^−/−^ mice compared with wt mice. This has previously been shown to contribute to TNF-mediated caspase-8 activation and injury in mice with hepatocellular cFLIP deficiency.^8, 23^ In line with these previous findings, liver injury was abolished in DKO mice carrying a deletion of JNK2 in addition to the hepatocyte-specific loss of cFLIP. These results point toward activation of JNK2 from hypoinsulinemia, which mechanistically drives liver cell injury in flip^−/−^ mice.

In summary, the loss of insulin in a mice susceptible to cellular injury from apoptosis leads to activation of the injurious cellular stress signaling kinase JNK2. The initiation of liver cell injury is accompanied by an increase in, especially, NK cells in the hepatic tissue and by an enhanced secretion of proinflammatory cytokines, both of which potentially contribute to the recruitment of inflammatory lymphocytes causing progressive cellular injury. Correction of metabolic abnormalities, inhibition of caspases and deletion of JNK2 or loss of B and T lymphocytes reverse this phenotype and prevent liver cell injury. Thus, we conclude that direct interactions between insulin, stress and apoptosis signaling pathways in hepatocytes exist, and the increasing prevalence of metabolic risk factors are potential modulators of cellular survival in the liver.

## Materials and Methods

### Animal models and serological analysis

Mice with a hepatocyte-specific deletion of cFLIP using the cre-loxP system under the control of the albumin promoter were used (flip^−/−^).^5^ Rag-1-deficient mice were obtained from Charles River, Jackson Laboratories (Wilmington, MA, USA). All animals were bred and maintained in the animal facility of the University Medical Center of the Johannes Gutenberg University (Mainz, Germany), according to the criteria outlined by the ‘Guide for the care and Use of Laboratory Animals' and approved by the Committee for Experimental Animal Research, and kept in a 12 h light/dark cycle under standard conditions with free access to water and food. For experiments, female mice were injected with 80 mg/kg STZ or sodium citrate intraperitoneally on five consecutive days. Blood glucose and ALT measurements were recorded repetitively from whole blood or serum. To prevent mortality, mice were injected with insulin glargin (Novo Nordisk, Mainz, Germany) to correct blood glucose levels when above 250 mg/dl. When indicated, a cellular suspension of 6 × 10^6^ bone marrow cells derived from rag-1-deficient mice was injected through the tail vein into 12- to 14-week-old wt or flip^−/−^ mice following lethal irradiation (2 × 3 Gy within 3 h). Further experiments were performed 4 weeks post transplantation. Blood glucose and ALT were determined from serum obtained by retro-orbital bleeding and analyzed using a standard clinical analyzer (Hitachi 917; Roche Diagnostics, Mannheim, Germany).

### Isolation of primary hepatocytes by collagen perfusion

Murine hepatocytes were isolated using two-step collagenase perfusion. Briefly, mice were anesthetized, the portal vein was intubated and the liver was perfused with a calcium-free buffer (0.14 M NaCl, 6.2 mM KCI, 10 mM HEPES at pH 7.4) at 37 °C. The perfusion was then switched to a collagenase solution (0.066 M NaCI, 6.7 mM KCI, 6.3 mM CaCl_2_, 0.1 M HEPES, 75 collagen digestion units of collagenase isoform type I at pH 7.6). After 10 min of perfusion with collagenase, the liver cells were dispersed, filtered, resuspended (0.068 M NaCI, 5.4 mM KCI, 1.1 mM KH_2_PO_4_, 0.7 mM Na_2_SO_4_, 1.4 mM MgCl_2_, 1.6 mM CaCI_2_, 30 mM HEPES, 0.03 M TES, 0.03 M Tricine at pH 7.6) and washed three times. Viability was assessed by Trypan blue exclusion and reached 90% in all subsequent experiments. Collagenase and chemicals were purchased from Sigma-Aldrich (Steinheim, Germany). Where indicated, cells were pretreated with the pancaspase inhibitor zVAD or SP600125 (both Calbiochem/Merck AG, Darmstadt, Germany), both dissolved in dimethyl sulfoxide (Sigma-Aldrich). Cell viability was assessed by neutral red staining. Briefly, cells were incubated for 2 h at 37 °C with medium containing neutral red (Sigma-Aldrich); they were then washed and absorbance was read using a spectrophotometer at 550 nm after extraction of the dye with 1.5 ml of *N*-propanol. The relative cell survival was calculated by dividing the optical density of a treatment group by the optical density of untreated control cells.

### Isolation of intrahepatic immune cells

Mice were anesthetized and the liver was perfused with 3 ml of RPMI 1640 medium (Life Technologies, Carlsbad, CA, USA) containing 0.5 mg/ml collagenase A (Roche Diagnostics) by intubation of the portal vein. The liver was then removed and mechanically disrupted 30 min before incubation in RPMI 1640 medium supplemented with 10% FCS, 0.5 mg/ml collagenase A and 5000 U/*μ*l DNase I (Roche Diagnostics) at 37 °C. The resulting tissue slurry was passed through a sterile, 70-*μ*m Falcon cell strainer (BD Biosciences, San Jose, CA, USA) and centrifuged three times at 500 r.p.m. for 5 min and without a break to remove hepatocytes. After centrifugation at 1600 r.p.m. for 7 min and without a break, erythrocytes were lysed with a hypotonic solution of ammonium chloride, and the lysed mixture was again centrifuged. The resulting cells were resuspended in PBS (pH 7.0) containing 2% FCS for further analysis.

### Quantitative real-time PCR

Total RNA was isolated using the TRI Reagent RNA isolation Kit (Sigma-Aldrich) according to the manufacturer's instructions. Equal amounts of total RNA were used to synthesize cDNA with the Affinity Script QPCR Kit (Stratagene, La Jolla, CA, USA). RTq-PCR was performed using SYBR Green, 96-well plates (Thermo Fisher Scientific, Schwerte, Germany) and the LightCycler 480 Real-Time PCR System (Roche Diagnostics). Real-time PCR was carried out with duplicate targets, controls and negatives. Wells were loaded with 1.5 *μ*l cDNA, 1 *μ*l Primer (Qiagen QuantiTect Primer Assays, Mm_Cflar_1_SG (QT00171738) or Mm_Gapdh_3_SG (QT01658692)), 1.5 *μ*l H_2_O and 5 *μ*l QuantiTect SYBR Green PCR Kit (Qiagen, Hilden, Germany), and hot-start PCR was performed with 50 cycles (denaturization for 15 s at 94 °C, annealing for 30 s at 55 °C and elongation for 30 s at 72 °C). Roche LightCycler software (LightCycler 480 Software Release 1.5.0 (Roche Diagnostics)) was used to perform advanced analysis relative quantification using the 2^(−ΔΔC(T))^ method.^28^ Expression data were normalized to the housekeeping gene *GAPDH*. The mean of untreated wt mice was considered 100%.

### Histological analysis and fluorescence microscopy

Histological examination was performed on paraffin-embedded sections after hematoxylin and eosin (H&E) staining. Blinded grading for inflammation using the Metavir score was performed by an experienced mouse histopathologist (AS). TUNEL was performed according to the manufacturer's instructions (Roche Diagnostics). Activated caspase-3 was quantified by fluorescence microscopy following staining with primary and Alexa Fluor 555-conjugated secondary antibodies. In short, paraffin-embedded sections were deparaffinized, rehydrated and stored in Na citrate solution for antigen retrieval. To reduce unspecific staining, sections were blocked for 2 h, stained with cleaved caspase-3 antibody (Cell Signaling Technology Inc., Danvers, MA, USA) overnight at 4 °C and visualized using Alexa Fluor 555-conjugated anti-rabbit secondary antibody (Cell Signaling Technology Inc.). Sections were DAPI-counterstained (Sigma-Aldrich) and prepared for microscopy with Kaisers glycerol gelatine (Merck, Darmstadt, Germany). Quantification of positively stained hepatocytes was performed from at least 40 visual fields at × 200 magnification and results are expressed as positive cells per visual field.

### Western blot and markers of oxidative stress

Western blotting was performed as described previously.^5^ Primary antibodies included the following: actin (Sigma-Aldrich), phospho-JNK, p100/52, phospho-p65, p65, phospho-Akt, total Akt, IRS-1 and phospho-serine 307 IRS-1 (all Cell Signaling Technology Inc.), and JNK (Santa Cruz Biotechnology, Santa Cruz, CA, USA). Membranes were exposed to goat anti-rabbit and anti-mouse secondary antibodies conjugated with horseradish peroxidase (Sigma-Adrich). Densitometry was performed using ImageJ software (National Institutes of Health, Bethesda, MD, USA). Malondialdehyde levels were determined from whole liver tissue according to the manufacturer's instructions (Lipid Peroxidation (MDA) Assay Kit; BioVision, Mountain View, CA, USA).

### Flow cytometric analysis

All antibodies used for flow cytometry analysis of intrahepatic immune cells were purchased from Biolegend (London, UK). Data were obtained on a BD FACS Canto II flow cytometer (BD Biosciences, Heidelberg, Germany) evaluating at least 1 million events per sample. Analysis was performed using FlowJo software (TreeStare, Olten, Switzerland). Concentrations of CCL2 (MCP-1), IFN-*γ* and TNF in the serum were measured using a CBA Mouse Inflammation Kit (BD Biosciences) and analyzed using FCAP Array v.3 Analysis software (Soft Flow, St Louis Park, MN, USA).

### Statistical analysis

All numerical results are expressed as mean±S.E. and represent data from a minimum of three independent experiments. Calculations were made with Sigma Plot 2000 (SPSS Science, Chicago, IL, USA) and statistical significance was ascertained using Student's *t*-test.

## Abbreviations

cFLIP: cellular FLICE-inhibitory protein
JNK: c-Jun N-terminal kinase
NASH: non-alcoholic steatohepatitis
STZ: streptozotocin
MAPK: mitogen-activated protein kinases
IRS: insulin receptor substrate
